# Gene Therapy Approach for Treatment of Obese Agouti Mice

**DOI:** 10.3390/ijms252212144

**Published:** 2024-11-12

**Authors:** Maxim A. Yunin, Stanislav S. Boychenko, Petr Lebedev, Alexey V. Deykin, Mikhail V. Pokrovskii, Alexander D. Egorov

**Affiliations:** 1 Center for Translational Medicine, Sirius University of Science and Technology, Olympic Ave. 1, 354340 Sirius, Russia; yuninma@gmail.com (M.A.Y.); bojchenko.ss@talantiuspeh.ru (S.S.B.); 2 Center for Preclinical and Clinical Research, Belgorod State National Research University, 85 Pobedy St., 308015 Belgorod, Russia; artkelt98@yandex.ru (P.L.); alexei@deikin.ru (A.V.D.); pokrovskii@bsu.edu.ru (M.V.P.)

**Keywords:** obesity, agouti mice, lipid profiling, TAG, mass spectrometry

## Abstract

Obesity is a significant metabolic disorder associated with excessive fat accumulation and insulin resistance. In this study, we explored a gene therapy approach to treat obesity in agouti mice using adeno-associated viruses (AAVs) carrying PRDM16, FoxP4, or Follistatin (FST) genes, which are known to promote the browning of white adipose tissue. Mice treated with AAVs encoding PRDM16, FoxP4, or FST genes showed a reduction in body weight (10–14%) within the first three weeks after administration, compared to the control groups. A lipidomic analysis of the adipose tissue revealed a dramatic reduction in triacylglycerol (TAG) species with low carbon numbers (40–54 acyl carbons) in treated mice.

## 1. Introduction

The ability to store lipids is basic for energy metabolism. Specifically, these high-energy molecules are stored in mammalian adipose tissue. Adipocytes are lipid-filled cells that exert many different roles, from energy storage to energy expenditure, depending on the adipocyte type [1]. There are three types of adipose tissue: white, brown, and beige adipose [2,3]. White adipose tissue (WAT) is the most abundant type of adipose in adults, and the occurrence of obesity is associated with WAT expansion. White adipocytes are non-thermogenic and contain unilocular lipid droplets that occupy more than 95% of the adipocyte cell volume [4]. Brown adipose tissue has a characteristic dark color due to the higher concentration of mitochondria. Mature brown adipocytes are characterized by thermogenin expression (uncoupling protein 1, UCP1). In mitochondria, UCP1 provides the transfer of protons to the matrix without ATP synthesis, resulting in the release of energy in the form of heat [5]. Brown and white adipocytes originate from different cell lineages [6,7]. However, it was revealed that the activation of the brown adipocyte differentiation program in white adipose tissue cells leads to conversion into another type of adipocytes. This process could be induced by various multiple stimuli, including chronic cold exposure [8], and leads to conversion into beige or “brite” (brown in white) adipocytes. These beige adipocytes are inducible thermogenic cells that occur as clusters within WAT depots, exhibiting a mixed energy storage or spending function and a mixed multilocular/unilocular/paucilocular morphology. Beige adipocytes are characterized by low basal expression of UCP1, which is inducible upon stimulation by cold or other stimuli [1,8]. Their response includes an increase in UCP1 expression, thermogenesis, and intensification of respiratory rate and lipolysis.

Obesity is a chronic disease characterized by excessive fat deposition, the primary cause of which is metabolic imbalance [9]. Animal models have been used extensively as tools to elucidate human obesity genes and mechanisms of human obesity. The *agouti* gene is involved in the regulation of melanin synthesis in wild-type mice. When the *agouti* is expressed in the hair follicle during early postnatal development, it switches the pigment synthesis from black (eumelanin) to yellow (phaeomelanin), resulting in a subapical yellow coloration [10]. Such dominant mutations of *agouti* as the lethal yellow (AYIa) and viable yellow (AVYIa) are characterized not only by their yellow coat color, but by a pleiotropic syndrome that includes increased body size, increased susceptibility to cancer, insulin resistance, and maturity-onset obesity [11]. Obese animals exhibit body fat mass that is 35–50% greater than wild-type mice, and this obesity is associated with elevated hepatic lipogenesis, depressed basal lipolysis, and adipocyte hypertrophy [12].

Nowadays, novel drugs that effectively overcome insulin resistance and counteract obesity are in development. Special attention is paid to viral and non-viral vectors targeting transcriptional pathways by the overexpression of genes encoding transcription factors controlling brown adipogenesis. Studies have demonstrated that activation of specific transcriptional regulators, such as PRDM16 (the transcriptional regulator PR domain-containing protein 16), PGC-1α, and C/EBPβ, and recently discovered FoxP4 (forkhead box transcription factor 4) can drive the browning process [3,4,13,14]. Here, we hypothesize that adeno-associated viral (AAV) delivery of PRDM16 or FoxP4 to agouti mice leads to weight loss and changes in the lipid profile of WAT. We found a decrease in weight after AAV administration. Additionally, we discovered that gene therapy application lowers the TAG content (up to a 50-fold decrease for some TAG species) in lipid extracts of WAT in agouti mice after PRDM16 or FoxP4-expressing AAV injection in comparison with the control agouti mice.

## 2. Results

### 2.1. Expression Cassette for AAV Production

**Figure 1:**
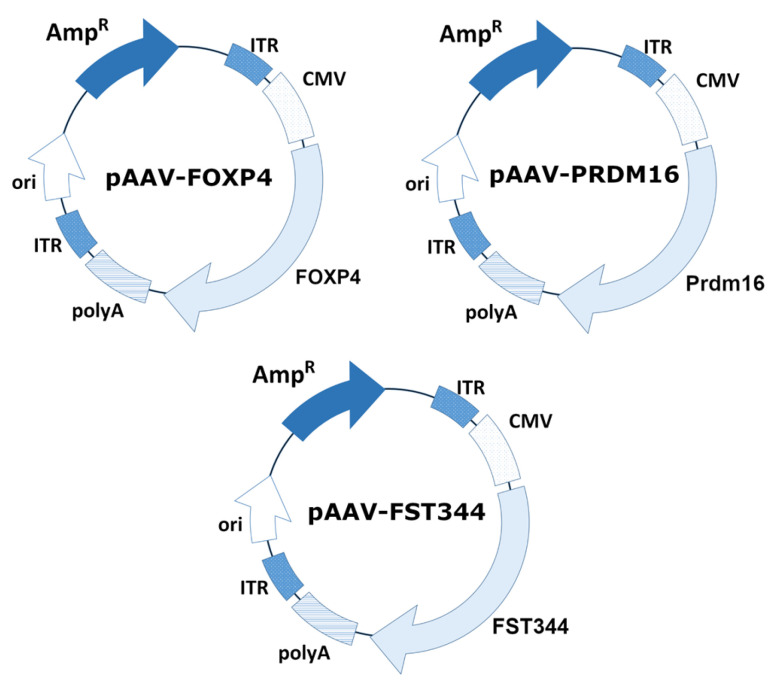
Schematic representation of plasmid map pAAV-FoxP4, pAAV-PRDM16, and pAAV-FST.

An AAV expression cassette was obtained by cloning the PRDM16, FoxP4, and FST genes between the ITRs into multiple cloning sites of the vector backbone pAAV-CMV-MSC. The schematic plasmid maps are presented in Figure 1. The mouse PRDM16 gene was amplified from a commercially available plasmid, pcDNA3.1 PRDM16 (Addgene plasmid #15503; RRID: Addgene_15503). The human FoxP4 gene was amplified from a commercially available plasmid, Flag-FoxP4 (Addgene plasmid #153148; RRID: Addgene_153148). The human FST was amplified from a cDNA obtained from HepG2 cells.

### 2.2. Production of Recombinant AAV8-PRDM16, AAV8-FoxP4, AAV9-FST

**Figure 2:**
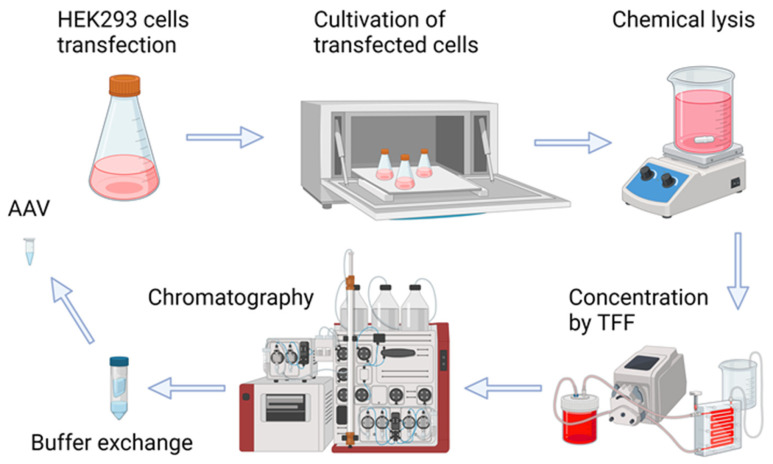
Schematic representation of adeno-associated virus production and purification.

The initial step in the study is the production of high-quality recombinant viruses and purification procedures (see Figure 2 for a representation of the process of recombinant AAV production). In summary, transfection of a suspension HEK293 cell culture with three plasmids (pAAV-FoxP4/pAAV-PRDM16/pAAV-FST, pHelper, and the corresponding pRC) resulted in the production of rAAV. Five days after transfection, the cells were lysed, and the resulting cell lysates, which contained rAAV particles, were subjected to concentration by tangential flow filtration. The concentrated virus was purified by chromatography using CaptureSelect AAVX affinity resin. Subsequently, ultrafiltration was performed to exchange the buffer and remove excess salt.

The produced rAAV samples were analyzed using dynamic light scattering (DLS). The results of the dynamic light scattering analysis demonstrated the presence of AAV8-PRDM16, AAV8-FoxP4, and AAV9-FST particles with hydrodynamic diameters of 26.92 nm, 32.08 nm, 27.49 nm, and 26.62 nm at a concentration of 1.39 × 10^12^, 3.2 × 10^13^, 2.38 × 10^13^, and 4.51 × 10^12^, respectively. The average volume fraction of particles was 99.99%.

Genomic titers of the rAAVs were determined in the range of 1 × 10^11^–1 × 10^13^ VG/mL (viral genomes per liter of culture), as determined by RT-qPCR. Total viral particle concentrations were 2.67 × 10^13^ (AAV8-PRDM16) and 6.67 × 10^12^ (AAV8-FoxP4).

### 2.3. Administration of AAV with Encoded Transcriptional Regulators into Agouti Mice Results in Weight Loss Within the First 3 Weeks

**Figure 3:**
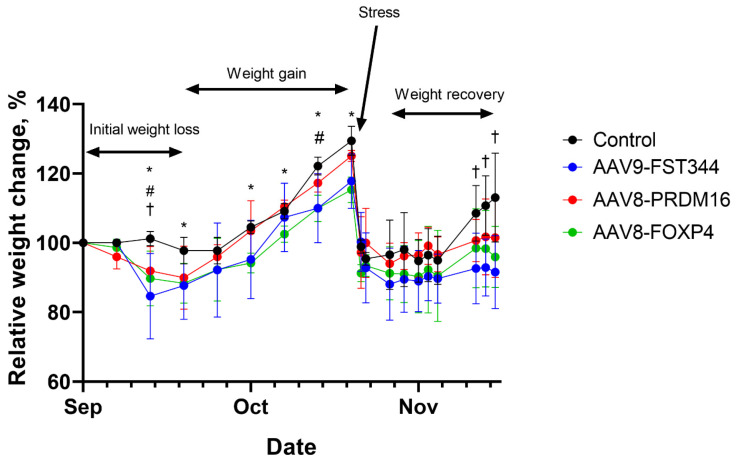
Progression of body weight change relative to weight before AAV administration in agouti mice treated at 12 weeks of age with an intra-WAT injection of empty AAV (Control), AAV-FoxP4, AAV-PRDM16, or AAV-FST vectors. Data are presented as mean ± S.D. values of three mice for each group. * *p* < 0.1 control group vs. AAV-FoxP4 group, # *p* < 0.1 control group vs. AAV-PRDM16 group, † *p* < 0.1 control group vs. AAV-FST group.

Obese agouti mice treated with AAV encoding human follistatin, FoxP4, or PRDM16 genes had significantly decreased weight gain (10–14%) within the first 3 weeks compared with levels prior to AAV administration, while there was no change in the control (empty AAV) group (Figure 3). Then, mice from all groups showed a marked increase in body weight gain. After 8 weeks, mice from all groups regain 25–30% of their initial weight (Figure 3). At the end of 8 weeks, the mice were transported from Belgorod to Sirius University to analyze the lipid composition of adipose tissue. Transport stress resulted in serious weight loss, mice from all groups showed similar weight loss of up to 40% of their pre-transport weights (Figure 3). After transportation, the mice treated with AAV-FoxP4, AAV-PRDM16, or AAV-FST vectors gained weight slower than the control animals (Figure 3).

### 2.4. AAV8-PRDM16, AAV8-FoxP4, and AAV9-FST Administration Cause Significant Reduction in the Content of TAG with Relatively Low Carbon Number (40–54 Acyl Carbones) in Subcutaneous Adipose Tissue

To explore lipids in iWAT that differed between mice that received FST, PRDM16, or FoxP4-expressing AAV vs. control (empty AAV), all of the significantly changed lipids were visualized using a volcano plot (Figure S1). Lipids that were altered by more than 2.0-fold with *p* < 0.05 were considered to be significantly altered between the two groups. Of the 168 lipids detected, 85 lipids showed significant differences in their abundance when mice received AAV9-FST and the controls were compared. The complete list of these lipids is shown in Table 1. For AAV8-FoxP4, 50 lipids, and for AAV8-PRDM16, 68 lipids were significantly altered compared to the control (Table 1). Photographic images of the adipose tissue that was used for the lipidomic analysis are shown in Figure S2.

Lipids that were similar across groups were identified and were compared based on fold changes. The comparison was made between the AAV9-FST, AAV8-FoxP4, and AAV8-PRDM16 groups against the control (Table 1). Based on these comparisons, we found that the levels of TAG with a relatively low carbon number (40–54 acyl carbons) and containing saturated or monounsaturated fatty acids were significantly decreased (up to 50-fold) after the administration of AAV9-FST, AAV8-PRDM16, or AAV8-FoxP4.

## 3. Discussion

The conversion of white to beige adipose tissue occurs naturally but can also be induced artificially in many ways. Cold exposure, dietary interventions, β3 adrenergic stimulation, and exercise training are some ways that lead to WAT browning and BAT activation [16,17,18,19].

PRDM16 is a dynamic transcriptional regulator of various stem cell niches that controls the development of brown adipocytes in BAT depots [20]. PPARγ, which stimulates adipocyte maturation, directly recruits PRDM16 to form a transcriptionally active complex that triggers a browning program in WAT. FoxP4 is expressed in adipose tissue and directly controls the levels of UCP1, a key regulator of thermogenesis that uncouples fatty acid oxidation from ATP production [5]. Recently, the overexpression of FoxP4 and PRDM16 was shown to affect thermogenic gene expression in in vitro cell-based models [13,20]. To our knowledge, this is the first study that uses AAV-mediated delivery of FoxP4 and PRDM16 genes in obese mice. We show that AAV-PRDM16 or AAV-FoxP4 administration induces loss of body weight in obese mice within the first three weeks after AAV administration and decreases iWAT lipid levels. The size of the iWAT of the AAV-FoxP4 and AAV-PRDM16 injections was smaller and darker than the controls (Figure S2). We also compare the efficacy of AAV-PRDM16 and AAV-FoxP4 with AAV-FST. FSTs have been demonstrated to play a key role in regulating white adipose browning both in in vitro and in vivo animal models [21]. FST functions to bind and neutralize the activity of follicle-stimulating hormone, activin, and members of the transforming growth factor-β superfamily [22]. Staining of the lipid droplets of the adipocytes demonstrated that the size of subcutaneous adipocytes in the FST-injected mice was significantly smaller than in the controls, and multilocular lipid droplet structures appeared in the FST injection group [22,23].

Using targeted MRM-based lipidomics, we found that AAV-FST, AAV-PRDM16, or AAV-FoxP4 injection decreased the overall abundance of the different lipid classes in iWAT. In the AAV-FST group, 20 phosphatidylcholines (PCs), 16 free fatty acids (FFAs), 4 cholesteryl esters (CEs), 11 sphingomyelins (SMs), 1 diacylglycerol (DAG), and 33 TAGs were significantly decreased compared to the controls, while only 2 PCs, 4 FFAs, 3 CEs, 2 DAGs, and 39 TAGs were significantly decreased in the AAV-FoxP4 group, and 12 PCs, 6 FFAs, 3 CEs, 5 SMs, 3 DAGs, and 39 TAGs were significantly decreased in the AAV-PRDM16 group (Table 1). TAG species with a low carbon number (40–54 acyl carbons) show the most dramatic decrease. Interestingly, it has been reported that TAG species with low carbon numbers and a low double-bond content have been associated with increased cardiovascular disease [24,25], suggesting that the AAV-FST, AAV-PRDM16, or AAV-FoxP4 injection may have beneficial effects in treating cardiovascular disease.

In this study, AAVs are directly injected into the fat pad of mice. Local AAV injections help mitigate off-target effects because of a lower dose and fewer leaks in the circulation. However, injecting fat depots is time-consuming and invasive. Systemic injection, such as intravenous and intraperitoneal injection, alternatively, could distribute AAVs in more fat depots, but it requires a higher dose than that of local injection and may provoke a greater immune response against the capsid and generate off-target effects [26,27]. We observed a 10% body weight reduction in the first 3 weeks after AAV administration and rapid weight regain during the next 5 weeks. We suppose that local injection of AAV-FST, AAV-PRDM16, or AAV-FoxP4 vectors do not efficiently target subcutaneous and visceral fat depots to alleviate obesity in mice for long periods of time.

We observed that mice treated with the AAV-FST vector nearly lost the ability to gain weight after transportation stress (Figure 3). Mice treated with the AAV-FoxP4 or AAV-PRDM16 vectors gained weight slowly after transportation stress (Figure 3). Further research in which data will be collected over a longer period of time is needed to confirm that the combination of AAV treatment and stress has synergistic effects.

## 4. Materials and Methods

### 4.1. Animal Procedures

Female agouti mice were bred at the Center for Preclinical and Clinical Research of Belgorod State National Research University (Belgorod, Russia) and taken for study at 12 weeks of age. All the animals with a body weight exceeding 28 g (12 mice) were randomly divided into four groups (3 mice per group) and given intro-inguinal white adipose tissue (iWAT) injections of control AAV (empty capsids), AAV-FoxP4, AAV-PRDM16, and AAV-FST [200 µL of each (2 × 10^10^ viral genomes in PBS)] on the left side. Mice were then fed a normal chow diet. The body weight was measured once a week after injection. Tissue sampling was performed after cervical dislocation. The expression of transgenes (FoxP4, PRDM16, FST) was not studied thoroughly as the adipose tissue samples were almost completely used for lipid analysis.

### 4.2. Sample Analysis Using Dynamic Light Scattering (DLS) and Size-Exclusion Chromatography (SEC) Methods

Dynamic light scattering (DLS) is a non-contact method that employs the light scattering effect and is designed to measure the size of nano- and submicron particles of a dispersed phase that exhibit Brownian motion. The DLS method offers a distinct advantage over other optical methods as it allows the sample to be measured in its native form. The samples were measured at 25 °C on a Zetasizer Ultra analyzer (Malvern Panalytical Ltd., Malvern, UK) equipped with a He–Ne laser with a wavelength of 633 nm and a maximum power of 10 mW. The multi-angle light scattering method, which is based on the sequential capture of the analytical signal from three detection angles of scattered radiation, permitted the estimation of the hydrodynamic diameter, the modality of particle distribution, and the fractional ratios. The Malvern Panalytical Ltd. (Malvern, UK) quartz cuvette was employed for the measurements. The data were processed using ZS XPLORER software, version 3.1.0 (Malvern Panalytical Ltd., Malvern, UK). The relative content of AAV monomers, high molecular weight substances (HMWS), and low molecular weight substances (LMWS) was determined by means of exclusion chromatography (SEC) using a Vanquish Flex liquid chromatograph (Thermofisher Scientific, Waltham, MA, USA). The fractions were separated in isocratic mode using an XBridge Protein BEH SEC chromatography column, 450 Å, 2.5 µm, 4.6 mm × 300 mm (Waters, Milford, MA, USA), with a mobile phase comprising. The solution was composed of 20 mM Na₂HPO₄, 150 mM KCl, and a pH of 7.0 with a flow rate of 0.5 mL/min. The fluorimetric detector was used for detection, with an excitation wavelength of 280 nm and an emission wavelength of 350 nm.

### 4.3. Transmission Electron Microscopy (TEM)

Transmission electron microscopy was employed for the observation of rAAV morphology. A total of 10 µL of viral suspension was applied to the freshly glow-discharged copper grids (200 mesh, formvar-carbon coated (EMCN, Beijing, China)), for 2 min, washed with distilled water, and stained with 1 droplet (10 µL) of a 1% (*w*/*v*) aqueous uranyl acetate solution (Polysciences Inc., Warrington, PA, USA, Catalogue No. 2024, 16, 138 5 of 14). The grids were observed using a transmission electron microscope, the JEM2100 Plus (JEOL, Tokyo, Japan), operating at 160 kV. A minimum of 15 grid squares were subjected to comprehensive examination, with representative micrographs captured at the same magnification.

### 4.4. Lipid Analysis

In total, 50 mg of adipose tissue was extracted with 500 µL of chloroform/methanol (2/1, *v*/*v*), centrifuged, and the supernatant was transferred to a vial and analyzed by liquid chromatography coupled with tandem MS (LC-MS/MS).

LC-MS/MS analysis was performed on a triple quadruple mass spectrometer (EVOQ Elite, Bruker, Bremen, Germany) coupled to an Ultimate 3000 RS UHPLC system (Thermo Scientific, Germering, Germany). The total run time was 65 min (Figure S3), and the flow rate was 0.21 mL/min. Chromatographic separation was achieved by reversed-phase chromatography and gradient elution. Separation of the lipids was carried out on a Poroshell 120 EC-C18 column (100 mm × 2.1 mm, particle size 1.9 μm, Agilent, Santa Clara, CA, USA). The injection volume was 1 µL. Column temperature was kept at 50 °C. Mobile phase A consists of isopropanol/methanol/water (5/1/4, *v*/*v*/*v*), with 5 mM ammonium acetate and 0.1% acetic acid, and mobile phase B consists of isopropanol/water (95/5, *v*/*v*), with 5 mM ammonium acetate and 0.1% acetic acid. The gradient starts at 100% A, held for 3 min, increasing to 20% B in 3–5 min, increasing to 30% B in 5–25 min, increasing to 98% B in 25–42 min, and keeping 98% B for 23 min. Subsequently, the column was re-equilibrated for 17 min at 100% A. The detection of lipids was performed in multiple reaction monitoring (MRM) modes using a triple quadrupole mass spectrometer equipped with a heated electrospray ionization (ESI) source. In total, 191 lipids were analyzed (Table S1), of which 163 lipids were identified in positive mode (CE, SM, Cer, TAG, DAG, PC) and 28 were identified in negative mode (FFA). All samples were measured in one run including positive and negative ion modes (Table S1, Figure S3). The source capillary voltage was 4400 and 3700 V in positive ion and negative ion modes, respectively. Cone temperature was set to 350 °C and cone gas to 20 psi. Heated probe temperature was set to 250 °C and probe gas flow to 30 psi. Nebulizing gas was set to 50 psi and collision gas (argon) to 1.5 mTorr.

MRM peak areas of lipids were calculated and submitted for statistical analysis. Bruker MS Workstation software (version 8.2.1; Bruker, Bremen, Germany) was used for mass spectrometry data acquisition and processing. Statistical analysis using peak areas was carried out by Metaboanalyst 5.0, https://www.metaboanalyst.ca/, (accessed on 12 August 2024) [15]. Fold change analysis was used to compare the lipid peak areas between mice who received control (empty AAV) or follistatin (FST), PRDM16, and FoxP4-expressing AAV.

## Figures and Tables

**Table 1:** The fold changes of the identified lipids between the AAV9-FST, AAV8-FoxP4, and AAV8-PRDM16 groups against the control (empty AAV). Fold change and *p*-value were calculated in Metaboanalyst 5.0 [15]. Lipids showing *p*-value > 0.05 are marked «Ns» (non-significant).

Compound	FoxP4/Control	PRDM16/Control	FST/Control
TAG (40:0/FA14:0)	0.020	0.021	0.037
TAG (40:0/FA16:0)	0.045	0.058	0.132
TAG (42:0/FA14:0)	0.080	0.106	0.129
TAG (42:0/FA16:0)	0.033	0.044	0.077
TAG (42:1/FA14:0)	0.041	0.045	0.067
TAG (42:1/FA16:0)	0.051	0.066	0.120
TAG (42:1/FA16:1)	0.044	0.046	0.069
TAG (42:1/FA18:1)	0.070	0.070	0.157
TAG (42:2/FA18:2)	0.093	0.107	0.203
TAG (44:0/FA14:0)	0.214	0.264	0.359
TAG (44:0/FA16:0)	0.144	0.150	0.187
TAG (44:0/FA18:0)	0.084	0.096	0.105
TAG (44:1/FA14:0)	0.260	0.324	0.331
TAG (44:1/FA16:0)	0.077	0.094	0.103
TAG (44:1/FA16:1)	0.216	0.230	0.275
TAG (44:1/FA18:1)	0.056	0.065	0.079
TAG (44:2/FA14:0)	0.176	0.174	0.149
TAG (44:2/FA16:0)	0.058	0.058	0.099
TAG (44:2/FA16:1)	0.132	0.122	0.131
TAG (44:2/FA18:1)	0.110	0.123	0.186
TAG (46:0/FA16:0)	0.174	0.237	0.300
TAG (46:1/FA16:1)	0.222	0.251	0.332
TAG (48:0FA 16:0)	0.250	0.403	0.392
TAG (48:0/FA 18:0)	0.147	0.202	0.316
TAG (48:1/FA 16:0)	0.204	0.255	0.363
TAG (48:1/FA 16:1)	0.138	0.196	0.345
TAG (48:1/FA 18:1)	0.337	0.370	0.408
TAG (48:2/FA 16:0)	0.430	Ns	Ns
TAG (48:2/FA 18:1)	Ns	Ns	0.404
TAG (48:2/FA 18:2)	0.404	Ns	Ns
TAG (50:0/FA 16:0)	0.212	0.368	Ns
TAG (50:1/FA 18:0)	0.292	0.417	Ns
TAG (50:2/FA 16:1)	0.276	0.397	Ns
TAG (52:0/FA 16:0)	0.228	0.298	0.199
TAG (52:0/FA 18:0)	0.111	0.159	0.240
TAG (52:1/FA 18:0)	Ns	0.482	Ns
TAG (52:2/FA 18:2)	0.358	0.368	Ns
TAG (52:4/FA 20:4)	Ns	0.249	Ns
TAG (52:5/FA 20:4)	0.375	0.159	Ns
TAG (54:0/FA 18:0)	0.214	0.331	0.337
TAG (54:1/FA 18:0)	0.190	0.294	0.304
TAG (54:1/FA 18:1)	0.316	0.415	0.385
PC (30:0)	0.398	0.301	0.168
PC (30:1)	Ns	Ns	0.323
PC (30:2)	Ns	Ns	0.342
PC (32:0)	Ns	Ns	0.287
PC (32:2)	Ns	0.427	0.292
PC (32:3)	Ns	Ns	0.313
PC (34:1)	Ns	0.458	0.279
PC (34:2)	Ns	0.488	0.343
PC (34:3)	Ns	Ns	0.357
PC (34:4)	0.395	0.439	0.281
PC (36:0)	Ns	Ns	0.458
PC (36:1)	Ns	0.492	0.433
PC (36:2)	Ns	Ns	0.499
PC (36:3)	Ns	Ns	0.357
PC (36:4)	Ns	0.335	0.262
PC (36:5)	Ns	0.318	0.292
PC (38:4)	Ns	0.369	0.378
PC (38:5)	Ns	0.407	0.240
PC (38:6)	Ns	0.307	0.289
PC (38:7)	Ns	0.272	0.295
SM (d16:1/18:1)	Ns	Ns	0.349
SM (d16:1/22:1)	Ns	Ns	0.315
SM (d16:1/24:0)	Ns	Ns	0.495
SM (d18:0/16:0)	Ns	Ns	0.350
SM (d18:0/18:0)	Ns	0.473	0.299
SM (d18:0/20:0)	Ns	0.450	0.272
SM (d18:0/22:0)	Ns	Ns	0.426
SM (d18:1/16:0)	Ns	Ns	0.341
SM (d18:1/18:0)	Ns	0.470	0.313
SM (d18:1/20:0)	Ns	0.449	0.241
SM (d18:2/24:1)	Ns	0.343	0.386
C14:0	0.366	Ns	0.442
C14:1	0.358	Ns	0.336
C16:0	Ns	Ns	0.345
C16:1	Ns	Ns	0.327
C18:0	Ns	Ns	0.390
C18:1	Ns	Ns	0.342
C18:2	Ns	Ns	0.445
C18:3	Ns	Ns	0.491
C20:1	Ns	Ns	0.412
C20:3	Ns	0.424	0.372
C20:4	Ns	0.480	Ns
C20:5	0.438	0.264	0.431
C22:1	Ns	Ns	0.354
C22:4	Ns	0.254	0.245
C22:5	0.402	0.205	0.289
C22:6	Ns	0.273	0.444
C24:1	Ns	Ns	0.376
DAG(14:0/18:1)	0.326	0.484	0.356
DAG(16:0/20:3)	Ns	0.491	Ns
DAG(16:0/20:4)	Ns	0.485	Ns
DAG(18:0/18:2)	0.484	Ns	Ns
16:0 Cholesteryl ester	0.245	0.281	0.477
16:1 Cholesteryl ester	0.273	0.242	0.392
18:0 Cholesteryl ester	Ns	Ns	0.439
18:1 Cholesteryl ester	0.438	0.452	0.418

## Data Availability

The data presented in this study are available on request from the corresponding author.

## Supplementary Materials

The following supporting information can be downloaded at: https://www.mdpi.com/article/10.3390/ijms252212144/s1.
