# Supplementary material for: Gene Therapy Approach for Treatment of Obese Agouti Mice
Source: Int J Mol Sci. 2024 Nov 12;25(22):12144. doi: 10.3390/ijms252212144 (PMC11594101; doi:10.3390/ijms252212144)
Supplement: Supplementary file 1 [file ijms-25-12144-s001.zip › ijms-3256965-supplementary.pdf]

Gene therapy approach for treatment of obese agouti mice

Maxim A. Yunin [1], Stanislav S. Boychenko [1], Petr Lebedev [2] and Alexander D. Egorov [1,*]

[1] Center for Translational Medicine, Sirius University of Science and Technology, Olympic Ave. 1, 354340 Sirius, Russia; yuninma@gmail.com (M.A.Y.); bojchenko.ss@talantiuspeh.ru (S.S.B.); egorov.ad@talantiuspeh.ru (A.D.E.)
[2] Center for Preclinical and Clinical Research, Belgorod State National Research University, 85 Pobedy St., 308015 Belgorod, Russia; artkelt98@yandex.ru
* Correspondence: egorov.ad@talantiuspeh.ru;

Supplementary Data

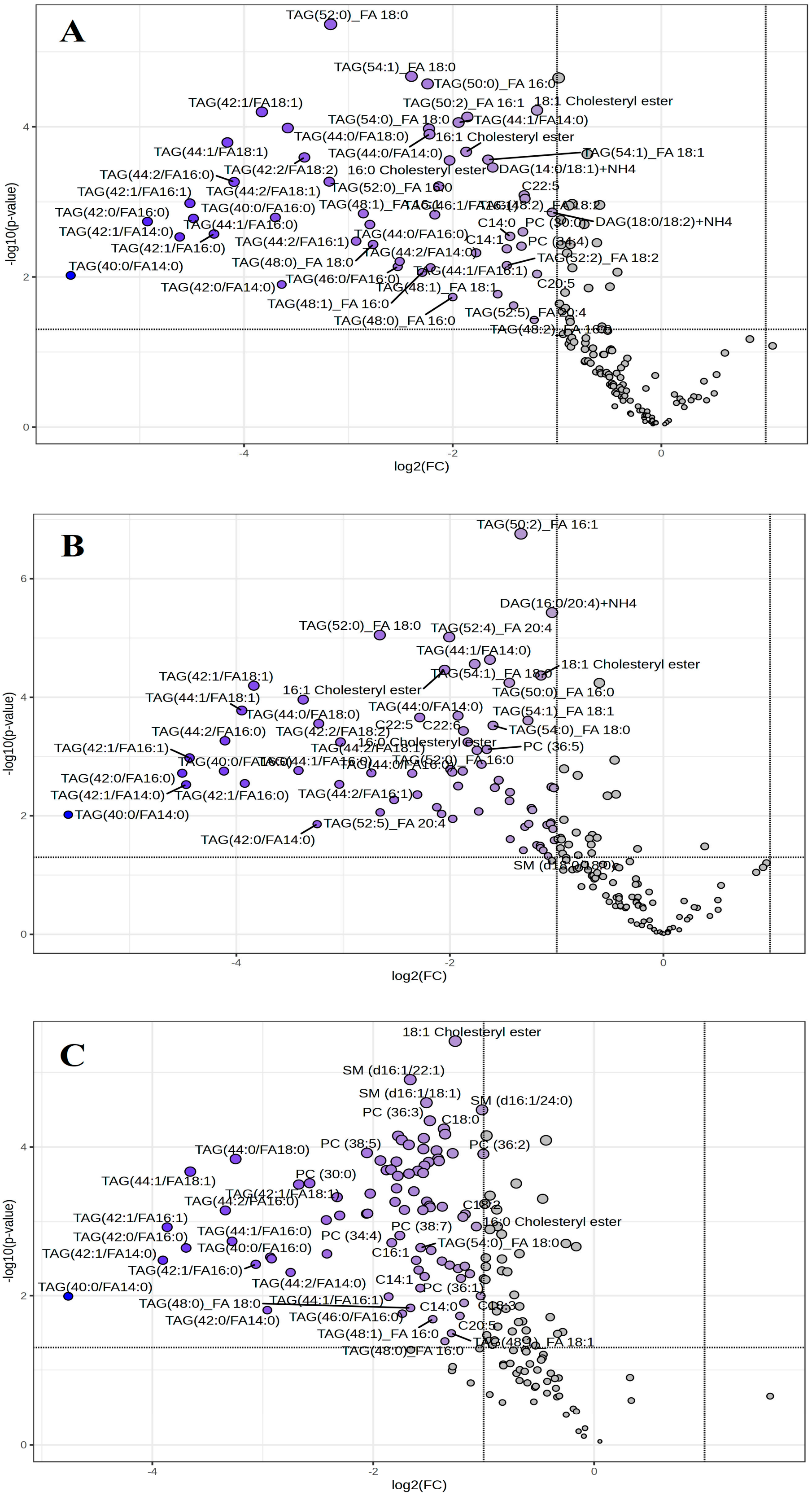

Figure S1. Volcano plots of significantly different lipids. Lipids that pass a threshold of fold change (FC) >2.0, p value < 0.05 are highlighted by blue and red. The highlighted by red lipids were increased and the highlighted by blue decreased in mice received FoxP4-expressing AAV (A), PRDM16-expressing AAV (B) or follistatin-expressing AAV (C) compared with the control (empty AAV). Volcano plot analysis was performed in Metaboanalyst 5.0 (https://www.metaboanalyst.ca/).

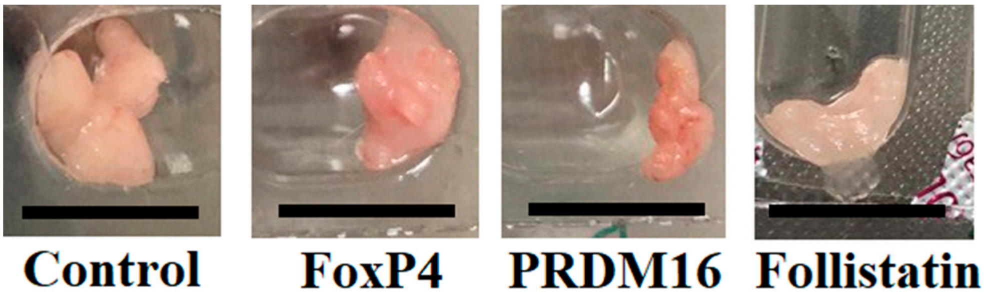

Figure S2. Photographic images of adipose tissue extracted from mice that were injected with empty AAV (control), AAV8-FoxP4 (FoxP4), AAV8-PRDM16 (PRDM16), and AAV9-FST (Follistatin). Scale bar: 10 mm.

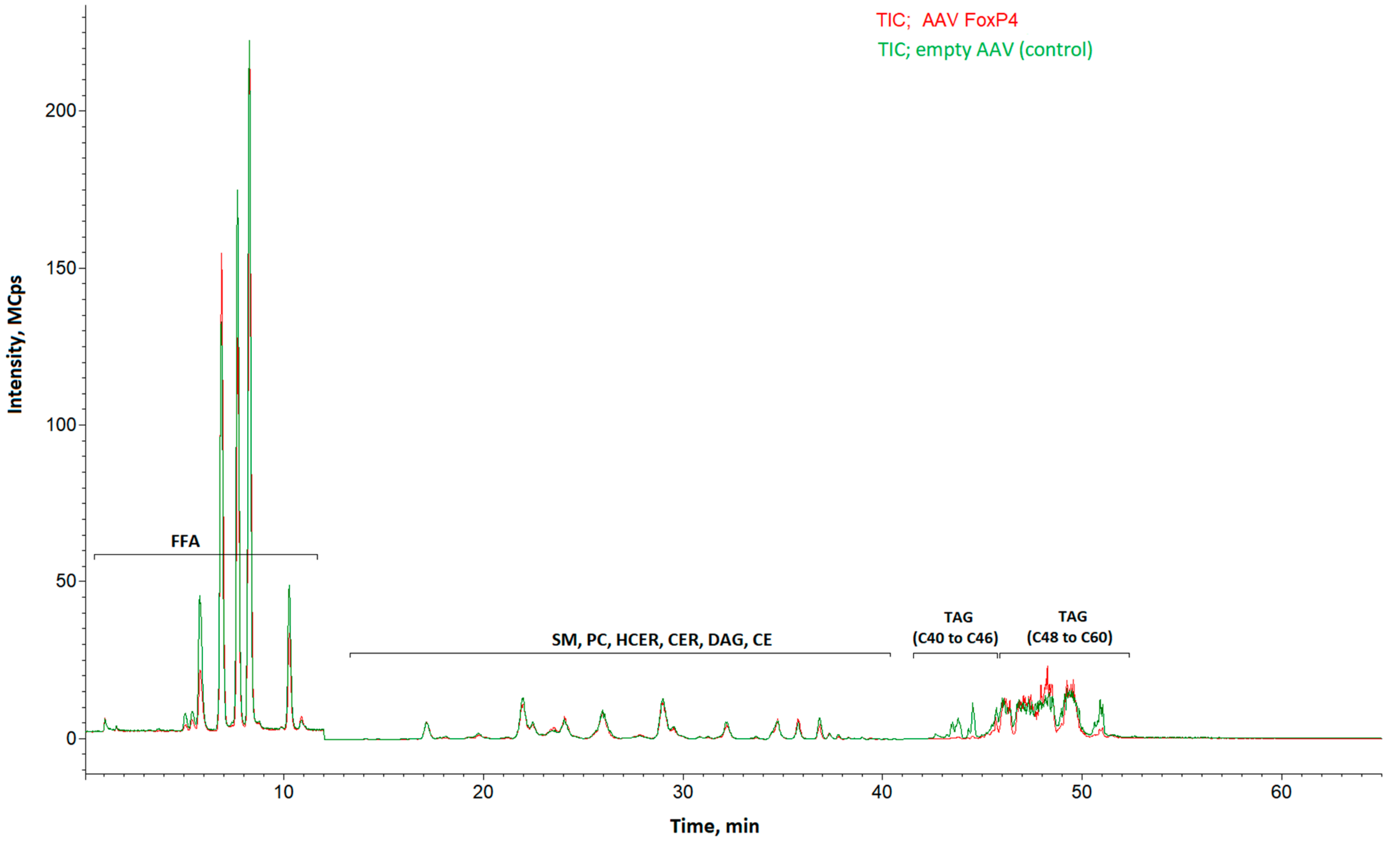

Figure S3. Total ion chromatogramms of iWAT lipids. Green: intra-WAT injection of empty AAV (control) and red: intra-WAT injection of AAV-FoxP4.

Table S1. MS/MS parameters of analyzed lipids.

Compound	Polarity	Precursor Ion (m/z)	Product Ion (m/z)	Time segment (min)
FFA C12:0	Negative	199.2	199.2	0-12
FFA C12:1	Negative	197.2	197.2	0-12
FFA C14:0	Negative	227.3	227.3	0-12
FFA C14:1	Negative	225.2	225.2	0-12
FFA C16:0	Negative	255.3	255.3	0-12
FFA C16:1	Negative	253.3	253.3	0-12

FFA C18:0	Negative	283.3	283.3	0-12
FFA C18:1	Negative	281.3	281.3	0-12
FFA C18:2	Negative	279.3	279.3	0-12
FFA C18:3	Negative	277.3	277.3	0-12
FFA C18:4	Negative	275.3	275.3	0-12
FFA C20:0	Negative	311.3	311.3	0-12
FFA C20:1	Negative	309.3	309.3	0-12
FFA C20:3	Negative	305.3	305.3	0-12
FFA C20:4	Negative	303.3	303.3	0-12
FFA C20:5	Negative	301.3	301.3	0-12
FFA C22:0	Negative	339.3	339.3	0-12
FFA C22:1	Negative	337.3	337.3	0-12
FFA C22:4	Negative	331.3	331.3	0-12
FFA C22:5	Negative	329.3	329.3	0-12
FFA C22:6	Negative	327.3	327.3	0-12
FFA C24:0	Negative	367.3	367.3	0-12
FFA C24:1	Negative	365.3	365.3	0-12
FFA C24:5	Negative	353.3	353.3	0-12
FFA C24:6	Negative	355.3	355.3	0-12
FFA C26:0	Negative	395.3	395.3	0-12
FFA C26:1	Negative	393.3	393.3	0-12
FFA C28:0	Negative	423.4	423.4	0-12
16:0 Cholesteryl ester	Positive	642.6	369.1	12-41
16:1 Campesteryl ester	Positive	654.6	369.1	12-41
16:1 Cholesteryl ester	Positive	640.6	369.1	12-41
16:2 Campesteryl ester	Positive	652.6	369.1	12-41
16:2 Cholesteryl ester	Positive	638.6	369.1	12-41
18:0 Cholesteryl ester	Positive	670.6	369.1	12-41
18:1 Campesteryl ester	Positive	682.6	369.1	12-41
18:1 Cholesteryl ester	Positive	668.6	369.1	12-41
18:2 Cholesteryl ester	Positive	666.6	369.1	12-41
SM (d16:1/18:1)	Positive	701.6	184.1	12-41
SM (d16:1/22:1)	Positive	757.6	184.1	12-41
SM (d16:1/24:0)	Positive	787.7	184.1	12-41
SM (d16:1/24:1)	Positive	785.6	184.1	12-41
SM (d18:0/16:0)	Positive	705.6	184.1	12-41
SM (d18:0/18:0)	Positive	733.6	184.1	12-41
SM (d18:0/20:0)	Positive	761.6	184.1	12-41
SM (d18:0/22:0)	Positive	789.7	184.1	12-41
SM (d18:1/16:0)	Positive	703.6	184.1	12-41

SM (d18:1/18:0)	Positive	731.6	184.1	12-41
SM (d18:1/18:1)9Z))	Positive	729.6	184.1	12-41
SM (d18:1/20:0)	Positive	759.6	184.1	12-41
SM (d18:1/24:0)	Positive	815.7	184.1	12-41
SM (d18:2/22:1)	Positive	783.6	184.1	12-41
SM (d18:2/24:1)	Positive	811.7	184.1	12-41
SM(24:1)	Positive	813.7	184.1	12-41
SM(26:0)	Positive	843.7	184.1	12-41
SM(26:1)	Positive	841.7	184.1	12-41
PC (30:0)	Positive	706.5	184.1	12-41
PC (30:1)	Positive	704.5	184.1	12-41
PC (30:2)	Positive	702.5	184.1	12-41
PC (32:0)	Positive	734.6	184.1	12-41
PC (32:2)	Positive	730.5	184.1	12-41
PC (32:3)	Positive	728.5	184.1	12-41
PC (34:0)	Positive	762.6	184.1	12-41
PC (34:1)	Positive	760.6	184.1	12-41
PC (34:2)	Positive	758.6	184.1	12-41
PC (34:3)	Positive	756.6	184.1	12-41
PC (34:4)	Positive	754.5	184.1	12-41
PC (36:0)	Positive	790.6	184.1	12-41
PC (36:1)	Positive	788.6	184.1	12-41
PC (36:2)	Positive	786.6	184.1	12-41
PC (36:3)	Positive	784.6	184.1	12-41
PC (36:4)	Positive	782.6	184.1	12-41
PC (36:5)	Positive	780.6	184.1	12-41
PC (38:1)	Positive	816.6	184.1	12-41
PC (38:2)	Positive	814.6	184.1	12-41
PC (38:3)	Positive	812.6	184.1	12-41
PC (38:4)	Positive	810.6	184.1	12-41
PC (38:5)	Positive	808.6	184.1	12-41
PC (38:6)	Positive	806.6	184.1	12-41
PC (38:7)	Positive	804.6	184.1	12-41
CER(16:0)	Positive	538.6	264.4	12-41
CER(18:0)	Positive	566.7	264.4	12-41
CER(18:1)	Positive	564.8	264.4	12-41
CER(20:0)	Positive	594.6	264.4	12-41
CER(22:0)	Positive	622.7	264.4	12-41
CER(24:0)	Positive	650.8	264.4	12-41
CER(24:1)	Positive	648.8	264.4	12-41

CER(26:0)	Positive	678.9	264.4	12-41
HCER(16:0)	Positive	700.7	264.4	12-41
HCER(20:0)	Positive	756.7	264.4	12-41
HCER(22:0)	Positive	784.9	264.4	12-41
HCER(24:0)	Positive	812.9	264.4	12-41
HCER(24:1)	Positive	810.9	264.4	12-41
DAG(14:0/18:1)	Positive	584.4	285.2	12-41
DAG(14:0/20:0)	Positive	614.6	285.2	12-41
DAG(16:0/18:0)	Positive	614.4	313.2	12-41
DAG(16:1/18:0)	Positive	612.6	311.3	12-41
DAG(16:0/18:1)	Positive	612.6	313.2	12-41
DAG(16:1/18:1)	Positive	610.4	311.2	12-41
DAG(18:0/18:2)	Positive	638.4	341.3	12-41
DAG(18:1/18:2)	Positive	636.5	339.3	12-41
DAG(16:0/20:3)	Positive	636.5	313.3	12-41
DAG(16:0/20:4)	Positive	634.5	313.3	12-41
DAG(18:2/18:3)	Positive	632.4	337.3	12-41
DAG(18:1/20:1)	Positive	666.6	339.3	12-41
TAG(40:0/FA14:0)	Positive	712.6	467.4	41-65
TAG(40:0/FA16:0)	Positive	712.6	439.4	41-65
TAG(42:2/FA18:2)	Positive	736.6	439.4	41-65
TAG(42:1/FA14:0)	Positive	738.7	493.4	41-65
TAG(42:1/FA16:0)	Positive	738.7	465.4	41-65
TAG(42:1/FA16:1)	Positive	738.7	467.4	41-65
TAG(42:1/FA18:1)	Positive	738.7	439.4	41-65
TAG(42:0/FA14:0)	Positive	740.7	495.4	41-65
TAG(42:0/FA16:0)	Positive	740.7	467.4	41-65
TAG(44:2/FA14:0)	Positive	764.7	519.4	41-65
TAG(44:2/FA16:0)	Positive	764.7	491.4	41-65
TAG(44:2/FA16:1)	Positive	764.7	493.4	41-65
TAG(44:2/FA18:1)	Positive	764.7	465.4	41-65
TAG(44:1/FA14:0)	Positive	766.7	521.5	41-65
TAG(44:1/FA16:0)	Positive	766.7	493.4	41-65
TAG(44:1/FA16:1)	Positive	766.7	495.4	41-65
TAG(44:1/FA18:1)	Positive	766.7	467.4	41-65
TAG(44:0/FA14:0)	Positive	768.7	523.5	41-65
TAG(44:0/FA16:0)	Positive	768.7	495.4	41-65
TAG(44:0/FA18:0)	Positive	768.7	467.4	41-65
TAG(46:1/FA16:1)	Positive	794.7	523.5	41-65
TAG(46:0/FA16:0)	Positive	796.7	523.5	41-65

TAG(48:2)_FA 16:1	Positive	820.8	549.8	41-65
TAG(48:2)_FA 18:1	Positive	820.8	521.8	41-65
TAG(48:2)_FA 18:2	Positive	820.8	523.8	41-65
TAG(48:2)_FA 16:0	Positive	820.8	547.8	41-65
TAG(48:1)_FA 16:0	Positive	822.8	549.8	41-65
TAG(48:1)_FA 18:1	Positive	822.8	523.8	41-65
TAG(48:1)_FA 16:1	Positive	822.8	551.8	41-65
TAG(48:0)_FA 16:0	Positive	824.8	551.8	41-65
TAG(48:0)_FA 18:0	Positive	824.8	523.8	41-65
TAG(48:0)_FA 18:2	Positive	824.8	527.8	41-65
TAG(50:4)_FA 16:0	Positive	846.8	573.8	41-65
TAG(50:4)_FA 18:1	Positive	846.8	547.8	41-65
TAG(50:4)_FA 18:2	Positive	846.8	549.8	41-65
TAG(50:4)_FA 16:1	Positive	846.8	575.8	41-65
TAG(50:3)_FA 16:0	Positive	848.8	575.8	41-65
TAG(50:3)_FA 18:1	Positive	848.8	549.8	41-65
TAG(50:3)_FA 18:2	Positive	848.8	551.8	41-65
TAG(50:3)_FA 16:1	Positive	848.8	577.8	41-65
TAG(50:2)_FA 16:1	Positive	850.8	579.8	41-65
TAG(50:2)_FA 18:0	Positive	850.8	549.8	41-65
TAG(50:2)_FA 18:1	Positive	850.8	551.8	41-65
TAG(50:2)_FA 16:0	Positive	850.8	577.8	41-65
TAG(50:0)_FA 16:0	Positive	852.8	579.8	41-65
TAG(50:1)_FA 18:0	Positive	852.8	551.8	41-65
TAG(50:1)_FA 18:1	Positive	852.8	553.8	41-65
TAG(52:5)_FA 20:4	Positive	870.8	549.8	41-65
TAG(52:4)_FA 16:0	Positive	872.8	599.8	41-65
TAG(52:4)_FA 18:1	Positive	872.8	573.8	41-65
TAG(52:4)_FA 18:2	Positive	872.8	575.8	41-65
TAG(52:4)_FA 20:4	Positive	872.8	551.8	41-65
TAG(52:3)_FA 16:0	Positive	874.8	601.8	41-65
TAG(52:3)_FA 16:1	Positive	874.8	603.8	41-65
TAG(52:3)_FA 18:1	Positive	874.8	575.8	41-65
TAG(52:3)_FA 18:2	Positive	874.8	577.8	41-65
TAG(52:2)_FA 16:0	Positive	876.8	603.8	41-65
TAG(52:2)_FA 16:1	Positive	876.8	605.8	41-65
TAG(52:2)_FA 18:0	Positive	876.8	575.8	41-65
TAG(52:2)_FA 18:2	Positive	876.8	579.8	41-65
TAG(52:2)_FA 18:1	Positive	876.8	577.8	41-65
TAG(52:1)_FA 16:0	Positive	878.8	605.8	41-65

TAG(52:1)_FA 18:0	Positive	878.8	577.8	41-65
TAG(52:1)_FA 18:1	Positive	878.8	579.8	41-65
TAG(52:0)_FA 16:0	Positive	880.8	607.8	41-65
TAG(52:0)_FA 18:0	Positive	880.8	579.8	41-65
TAG(52:0)_FA 18:1	Positive	880.8	581.8	41-65
TAG(54:7)_FA 18:0	Positive	892.7	591.7	41-65
TAG(54:7)_FA 18:1	Positive	892.7	593.7	41-65
TAG(54:4)_FA 18:2	Positive	900.8	603.8	41-65
TAG(54:4)_FA 18:1	Positive	900.8	601.8	41-65
TAG(54:3)_FA 16:0	Positive	902.8	629.8	41-65
TAG(54:3)_FA 18:0	Positive	902.8	601.8	41-65
TAG(54:3)_FA 18:2	Positive	902.8	605.8	41-65
TAG(54:3)_FA 18:1	Positive	902.8	603.8	41-65
TAG(54:2)_FA 16:0	Positive	904.8	631.8	41-65
TAG(54:2)_FA 18:0	Positive	904.8	603.8	41-65
TAG(54:2)_FA 18:1	Positive	904.8	605.8	41-65
TAG(54:1)_FA 16:0	Positive	906.8	633.8	41-65
TAG(54:1)_FA 18:0	Positive	906.8	605.8	41-65
TAG(54:1)_FA 18:1	Positive	906.8	607.8	41-65
TAG(54:0)_FA 18:0	Positive	908.9	607.9	41-65
TAG(56:3)_FA 18:1	Positive	930.8	631.8	41-65
TAG(58:3)_FA 18:1	Positive	958.9	659.9	41-65
TAG(60:2)_FA 18:1	Positive	988.9	689.9	41-65
TAG(60:2)_FA 20:0	Positive	988.9	659.9	41-65
TAG(60:0)_FA 20:0	Positive	992.9	663.9	41-65
